# Healthcare interventions to improve health outcomes for racially/ethnic minoritised people with multiple long-term conditions: a systematic review and narrative synthesis

**DOI:** 10.1186/s12889-026-28056-y

**Published:** 2026-06-19

**Authors:** Brenda Hayanga, Mengxing Joshi, Kathryn Hartley, Patrick Oyibo, Rida Sarwar, Sakib Tariq, Mavin Kashyap, Laia Bécares

**Affiliations:** 1 Department of Global Health & Social Medicine, Faculty of Social Science & Public Policy, King’s College London, Bush House NE Wing, 30 Aldwych, London, WC2B 4BG UK; 2 School of Geography & Sustainable Development, University of St. Andrews, Fife, UK; 3 Social Research Institute, Institute of Education, University College London, London, UK; 4 School of Health & Medical Sciences, City St. George’s, University of London, London, UK; 5 Centre for Academic Primary Care, Bristol Medical School, Population Health Sciences, University of Bristol, Bristol, UK

**Keywords:** Race, Ethnicity, Inequalities, Multiple long-term conditions, Healthcare, Interventions

## Abstract

**Background:**

Racially/ethnic minoritised people with multiple long-term conditions (MLTCs) face inequalities across different dimensions of healthcare, yet little is known about how to improve their healthcare outcomes. This systematic review and narrative synthesis seeks to identify and describe healthcare interventions designed to improve health outcomes for racially/ethnic minoritised people with MLTCs and identify areas for further exploration. Given that primary care is considered the ideal setting to manage MLTCs, we focus on interventions targeted at healthcare providers/systems.

**Methods:**

We searched 9 bibliographic databases and one website and identified 6566 studies, 15 of which met the inclusion criteria. Given the heterogeneity of interventions, health conditions and outcomes of interest, we conducted a narrative synthesis.

**Results:**

The studies were conducted in the US (*n* = 13), Canada (*n* = 1) and Australia (*n* = 1). Most studies recruited racially/ethnic minoritised people mainly of African American and Hispanic/Latinx descent with comorbid Depression and a physical condition (Diabetes (*n* = 3), Hypertension (*n* = 3), Cancer (*n* = 2). Depression/mental health outcomes, patient-reported outcomes, clinical outcomes, medication use, and adherence were the most frequently assessed outcomes. Few studies reported on provider-related outcomes. All interventions made socio-cultural adaptations, thereby, promoting equitable and inclusive care. Community actors/assets were considered key to improving health outcomes. Of the 15 interventions, five resulted in statistically significant improvements in all outcomes of interest and nine resulted in improvements in some outcomes.

**Conclusions:**

This review illustrates the feasibility of socio-culturally adapted interventions, many of which successfully integrate physical and mental healthcare, delivered through multidisciplinary teams working collaboratively, and leveraging community assets to improve health outcomes for racially/ethnic minoritised people with MLTCs. Future research is needed to assess the impact of these interventions beyond North America and Australia. Studies are also required to identify provider-related outcomes with the potential to improve outcomes for racially/ethnic minoritised people with MLTCs.

**Supplementary Information:**

The online version contains supplementary material available at 10.1186/s12889-026-28056-y.

## Introduction

### Racial/Ethnic inequalities in multiple long-term conditions

People with two or more long-term conditions (MLTCs) have a poorer quality of life, a higher treatment burden, and an increased risk of polypharmacy, disability, and mortality compared to people with one long-term condition or none [1]. Studies suggest that many people from racially/ethnic minoritised^1^ groups have as many or more long-term conditions when compared to their white majority counterparts [2]. In the United States (US), for instance, analysis of data from nearly 600,000 people by Caraballo, Herrin [3] suggests that amongst participants aged 30 years and older, the prevalence of MLTCs among Black people is similar to that of Hispanic/Latino, white and Asian people who are 5–10 years older. These inequalities are also evident in the United Kingdom (UK) as the early onset of MLTCs has been found to be more common among South Asian and Black people when compared to white people [4]. Relatedly, an examination of age-related patterns of MLTCs across racial/ethnic groups suggests that racial/ethnic inequalities in MLTCs emerge from midlife onwards and by later life, older people of Pakistani, Indian, Black Caribbean and other ethnicity have more long-term conditions than white people [5]. The findings also suggest that compared to their white British counterparts, Gypsy or Irish Travellers are more likely to report MLTCs across all age groups [5].

When we consider other domains of health, including healthcare and mortality, studies show that racially/ethnic minoritised people with MLTCs receive poorer care quality, and have lower levels of satisfaction with primary care service than their white counterparts [6–8]. They also experience a higher risk of early death [9]. These inequalities are concerning and suggest that racially/ethnic minoritised people are disadvantaged at all stages of their MLTCs journey. Left unchecked, inequalities in MLTCs are likely to intersect with other forms of disadvantage to (re)produce new forms of inequality that will continue to negatively impact racially/ethnic minoritised people.

**Fig. 1:**
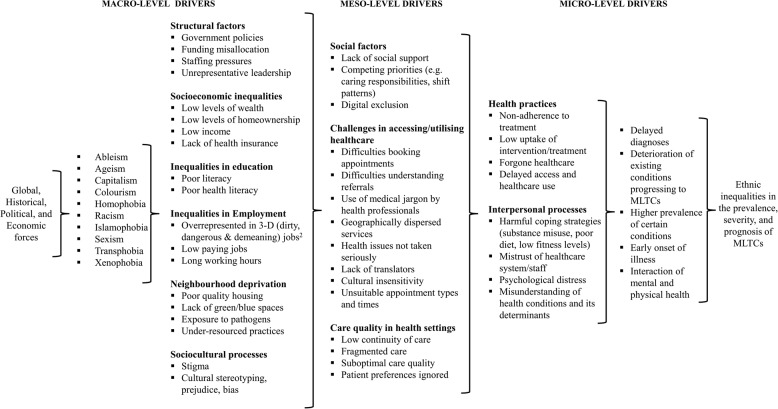
Drivers of racial/ethnic inequalities in multiple long-term health conditions 2

The inequalities outlined above raise questions regarding the drivers of racial/ethnic inequalities in MLTCs and the types of initiatives required to prevent the development of long-term conditions, progression towards MLTCs and, ultimately, improve outcomes for racially/ethnic minoritised people with MLTCs. Figure 1 depicts the drivers of racial/ethnic inequalities in MLTCs. It is based on evidence from the extant literature on racial/ethnic inequalities in MLTCs [6, 10] and informed by the works of Curtis, Paine [11], Hankivsky [12] and Bécares, Shaw [13]. The figure alerts us to the distal drivers of inequalities (e.g. racism) which intersect in complex ways with other powers of oppression (e.g. capitalism) to drive structural inequalities (e.g. allocation of resources, area deprivation, socioeconomic status). Not only do these inequalities impact negatively on healthcare provision, and care quality, but they also (in)directly affect health-seeking behaviour, healthcare utilisation and social support. In turn, these processes lead to the more proximal drivers of health inequalities (e.g. non-adherence, psychological distress, forgone healthcare, and delayed diagnosis), all of which contribute to racial/ethnic inequalities in the prevalence, severity, and prognosis of MLTCs. It is imperative that we identify and critically assess the types of interventions that have been designed and implemented to address racial/ethnic inequalities in MLTCs, the extent to which these interventions have been effective in improving health outcomes for racially/ethnic minoritised people with MLTCs and the mechanisms by which they do so.

### Interventions to improve health outcomes for people with MLTCs

Interventions to improve health outcomes for people with MLTCs are often based on models of care such as care planning, case management, collaborative care, integrated care, provider/information continuity, multidisciplinary working, medication management, and self-management [14]. Whether in combination or on their own, these interventions can be targeted at patients, providers, micro-systems, communities, organisations and/or policy [15]. In this review, we focus on interventions targeted at health providers/systems (situated at the meso-level in Fig. 1). Our decision is based on the premise that (a) interventions that narrowly focus on patient behaviour change can exacerbate existing inequalities [16], and (b) primary care is well suited to managing MLTCs since generalists and multidisciplinary teams can identify, manage, coordinate care and refer patients with the most prevalent health conditions [14, 17, 18]. Focusing on interventions targeted at health providers/systems shifts focus from the individual to healthcare provision. This is in line with recommendations by Clarke, Goddu [15] whose review of inequalities research over three decades found that research in this field has predominantly focused on the patient as the target for change. For Clarke, Goddu [15], research that investigates improvements in health systems that serve minoritised patients is crucial for addressing long-standing inequalities in health. This shift from patient to provider/system also aligns with recommendations by Chouhan and Nazroo [19] who advocate for a move beyond discourses that depict racial/ethnic inequities in health as a result of culture and genetics and instead focus on reforming institutional cultures, and social, economic and environmental accounting within healthcare systems.

To our knowledge, few systematic reviews have described and evaluated healthcare interventions targeted at healthcare providers/systems (henceforth, healthcare interventions) to improve health outcomes for people with MLTCs specifically. Those that have done so, have described and examined the impact of healthcare interventions for a range of outcomes for people with MLTCs. Examples of the types of healthcare interventions assessed in these reviews include interventions targeting the care delivered by primary care professionals [20], primary care teams [18], nurse-led interventions [21], and patient engagement interventions [22] for people with MLTCs. Collectively these reviews suggest that nurse-led interventions are of value to patients and healthcare systems [21] and that primary care teams can improve mental and psychological health outcomes [18]. Similarly, the majority of patient-engagement interventions identified by Søgaard, Andresen [22] led to higher quality-adjusted life-years, fewer hospital visits and disease specific symptoms. Mixed findings were reported by Smith, Wallace [20] whose review of professional, financial, organisational, regulatory and patient oriented interventions found that some interventions led to improvements in mental health outcomes, medication use and adherence, patient-reported outcomes, patient and provider health behaviour. However, they found little or no improvement in clinical outcomes and health service use [20].

Whilst much can be gleaned from existing reviews of healthcare interventions targeted at healthcare providers/systems to improve health outcomes for people with MLTCs, the focus of these reviews has not been on racially/ethnic minoritised people who collectively face multiple intersecting inequalities across the life-course, thereby, increasing their vulnerability to poor health and socioeconomic outcomes [23]. One review of team-based care for Cancer survivors with comorbidities identified 13 studies, four of which focused on Hispanic or Black people [24]. The authors highlight the lack of studies that concurrently assess care delivery and outcomes for Cancer and comorbidities. Owing to the narrow focus on team-based care and Cancer survivors with additional conditions, evidence of the types of healthcare interventions that can improve healthcare outcomes for racially/ethnic minoritised people with MLTCs is incomplete.

### Aims

This study aims to address the gaps in knowledge around effective interventions to prevent and/or improve the health outcomes of racially/ethnic minoritised people with MLTCs by systematically reviewing and synthesising evidence of healthcare interventions aimed at improving healthcare outcomes for racially/ethnic minoritised people with MLTCs. Specifically, we will identify and describe healthcare interventions targeted at healthcare providers/systems designed to improve health outcomes for racially/ethnic minoritised people living with MLTCs. To address this aim, we will answer the following research questions.

1. Which healthcare interventions have been implemented to improve health outcomes among racially/ethnic minoritised people with MLTCs?
2. What are the characteristics of healthcare interventions aimed at improving health outcomes for racially/ethnic minoritised people with MLTCs?
3. Which long-term conditions and outcome measures of interest are assessed?
4. To what extent do these interventions lead to improvements in health outcomes for racially/ethnic minoritised people?
5. What is the quality of evidence?

## Methods

### Search strategy

We registered the protocol of this systematic review and narrative synthesis on Prospero (CRD42023396657) [25] in accordance with guidance from the Preferred Reporting Items for Systematic review and Meta-analysis protocols (PRISMA-P)[26]. To identify relevant studies, we searched ASSIA, (Applied Social Sciences Index and Abstracts), Cumulative Index to Nursing and Allied Health Literature, MEDLINE, Journal of Health Visiting, PubMed, PsycINFO, Open Grey, National Grey Literature Collection, the International Research Community on Multimorbidity and Advancing Health Equity database^3^. An initial search was conducted in February 2023 by MJ and KH. We supplemented the electronic search with a manual search of key systematic reviews identified during screening. To ensure recent studies were not excluded, another search was conducted in August 2024 by BH and RS. This was followed by a search for studies linked to the included studies in November and December 2024 by BH and ST. The search was updated again in August 2025 by BH.

We formulated search terms based on five key concepts of the review i.e. Healthcare (e.g. health personnel OR health professional OR healthcare team), Intervention (e.g. program* OR case OR Randomi*ed controlled trial” OR “Controlled clinical trial” OR “Clinical trial”), Ethnicity (e.g. "Ethnic Groups"[MeSH) OR minorit* OR “BAME”), Inequalities (e.g. "Health Equity"[MeSH) OR “Healthcare disparit*” [MeSH) OR Inequalit* OR disparit*) and multiple health conditions (e.g. “Multiple Chronic Conditions” OR Multimorbidity OR Comorbid*) (See Supplementary Material 1 for a complete list of search terms and sample search strategy). We tailored the search to the conventions of each search engine and did not restrict the search to any period. We imported the studies retrieved from the electronic search to EPPI Reviewer 6 [27] for de-duplication, screening, quality assessment and data extraction.

### Eligibility criteria

We included studies published in English and conducted in countries with membership to the Organisation for Economic Co-operation and Development (OECD). Whilst these countries have different healthcare systems, they are all considered developed countries with high-income economies and are increasingly racially/ethnically diverse. The integration of racially/ethnically diverse populations into society is a key issue in public discourse [28] which can shape the health and socioeconomic outcomes for racially/ethnic minoritised people.

Our focus on interventions targeted at healthcare providers/systems meant that we excluded interventions that only seek to change health behaviour of racially/ethnic minoritised people with MLTCs. Among studies of healthcare interventions, we included studies reporting on outcomes for racially/ethnic minoritised people with MLTCs. Given the definition of MLTCs being the existence of two or more long-term conditions, we included studies of co-morbidity, where an index condition was specified and studies of multimorbidity, where no index condition was specified.

The screening team consisted of BH, MJ, KH, and RS. A random sample (10%) of the titles, abstracts, and full texts were double screened by BH and each member of the screening team. After reconciliation, each member was assigned a set of studies to screen independently. Double screening was conducted at different intervals to improve interrater reliability. Any differences were resolved through discussion during the reconciliation process with LB on hand to provide input in the event of any disagreements or if clarification was required.

### Data extraction

Data extraction was conducted by BH and RS using EPPI Reviewer 6 [27]. A random sample of the included studies (10%) were extracted by both BH and RS. Disagreements were settled by discussion. BH independently extracted data from the remaining studies. From the included studies, we extracted information on the study characteristics (e.g. authors, year of publication, study aim), participants characteristics (e.g. sample size, age, gender, ethnicity, health conditions), intervention characteristics (type, recruitment procedures, theoretical underpinning, setting, geographical location, health provider, socio-cultural adaptations, mechanisms underlying success of intervention), comparison intervention characteristics (e.g. usual care, enhanced usual care, none), outcomes assessed, key results and author interpretations.

### Quality assessment

The quality of the randomised controlled trials was assessed by BH, RS and ST using Cochrane Risk of Bias tool available in EPPI Reviewer 6 [27] (See Supplementary Material 2 for the risk of bias domains). BH assessed and jointly discussed one study with RS and another with ST . Thereafter, RS and ST individually assessed a subset of the remaining studies. The quality of the before-after studies was assessed using the National Heart Lung and Blood Institute [29] quality assessment tool for before-after studies (Supplementary Material 3). The studies were assessed for clarity in the objectives, participant selection criteria and representativeness, sample size adequacy, sufficiency of intervention description, validity of outcomes measures, blinding of participants/personnel, follow up, appropriate statistical analysis. BH and ST independently assessed the quality of one of these studies and discussed discrepancies. Subsequently, BH assessed the quality of the remaining studies. All studies, regardless of quality were included in the review given that the main objective was to identify and describe healthcare interventions for racially/ethnic minoritised people with MLTCs.

### Outcomes

The outcomes of interest were patient outcomes or health provider outcomes/processes. We were also interested in changes in these outcomes before and after implementation of the intervention, as reported by the authors.

### Data synthesis

There was heterogeneity in the intervention type, population, number of health conditions counted, and outcomes of interest. Consequently, we conducted a narrative synthesis of the findings [30]. The extracted data were read and re-read to identified patterns in the data which were synthesised and categorised into themes and subthemes. We present the results in three sections. First, we provide an overview of the studies identified and describe the patient and intervention characteristics of the healthcare interventions that sought to improve health outcomes for racially/ethnic minoritised people with MLTCs. Second, we present the impact of the interventions on outcome measures of interest. Finally, we present our appraisal of the quality of evidence from the studies included in this review. We supplement the reporting with tables and figures.

## Results

**Fig. 2:**
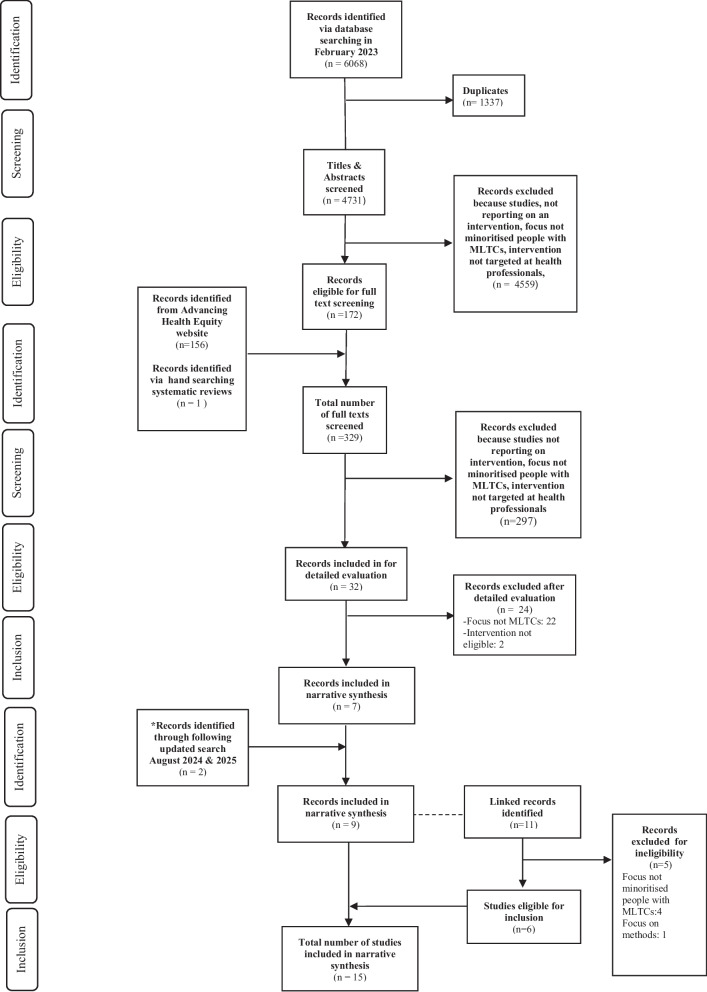
PRISMA Flow of Studies. (*See Supplementary Material 4 for the PRISMA Flow of Studies for the 2024 and 2025 searches)

We conducted an initial search in 2023 and additional searches in 2024 and 2025 to identify new studies (See Supplementary Material 4 for PRISMA Flow of Studies in 2024 and 2025). We identified 6068 studies from the first electronic search (Fig. 2). 1337 duplicates were removed leaving 4731 studies which were screened for eligibility using the title and abstract. Of these studies, 172 studies were deemed eligible for full-text screening. We identified an additional 156 studies from the Advancing Health Equity website which were included for full-text screening owing to their focus on healthcare interventions for racially/ethnic minoritised people with chronic conditions. An additional study was identified from existing systematic reviews. We screened 329 full texts and included 32 records for further evaluation. Of these, seven met the inclusion criteria. Following the 2024 and 2025 searches, we identified a further two studies eligible for inclusion. We manually searched for studies linked to the included studies using the authors’ names and identified 11 additional studies, six of which met the inclusion criteria. In total, 15 studies were included in the narrative synthesis.

### Characteristics of included studies

#### Geographical location, setting, duration, mode of delivery, follow up

The characteristics of the 15 studies included in this review are summarised in Table 1. The studies were published between 2003 and 2024. All but two studies were conducted in the US with one conducted in Australia [45] and another in Canada [44]. Of the 15 studies included, the most common study design was the Randomised Controlled Trial (RCT) (*n* = 9) [33, 37, 38, 40, 44], and pilot randomised controlled trials [31, 32, 41, 42] or a variant of the RCT (*n* = 2) e.g. pilot trial [34], and randomised pilot trial [36]. The remaining four studies were categorised as pre-test/post-test studies [35, 39, 43, 45]. The majority of studies (*n* = 11) were conducted in clinical settings, i.e. primary care practices (*n* = 5) [31–34, 41], public sector oncology clinics (*n* = 2) [36, 37], safety-net clinic (*n* = 1) [38], ambulatory setting (*n* = 1) [42], hospitals (*n* = 1) [45] and a free clinic embedded in a commercial centre [35]. Non-clinical settings included the community (*n* = 3) [39, 40, 44] and a specialised school setting (*n* = 1) [43]. There was variation in the duration of the interventions with the shortest intervention consisting of one visit [42] and the longest intervention running for 48 months [35]. Most interventions were delivered both in person and remotely (*n* = 10) and five interventions were delivered in person [34, 42–45]. There was also variation in assessment periods with participants assessed at two [35, 39, 45], three [33, 34, 36, 37, 41, 42], and four or more timepoints [31, 32, 38, 40, 43, 44].

**Table 1:** Study, Intervention and patient characteristics

Study ID	1 st Author Year of Publication	Study design	Country	Setting	Health conditions	Recruitment strategy	Mode of delivery	Duration	Follow up periods	Ethnicity as reported by authors	Age in years Mean (SD)	%Female	IntG (n)	ComG (n)	% in final analyses
[31]	Bogner (2008)	Pilot Randomised controlled trial (RCT)	USA	Primary Care practice	Depression & Hypertension	Upcoming appointments records	In-person, Phone	4 weeks	0, 2, 4, 6 weeks	81.2% African-American; 17.2% white; 1.6% Other	58.6 (6.8)	77	32	32	100
[32]	Bogner (2010)	Pilot RCT	USA	Primary Care practice	Depression & Diabetes	Upcoming appointments records	In-person, Phone	4 weeks	0, 2, 4, 6,12 weeks	100% African-American	Range: 50–58	84	29	29	100
[33]	Bogner (2012)	RCT	USA	Community-based primary care practices	Depression & Diabetes	Upcoming appointments records	In-person, Phone	3 months	0,6,12 weeks	56.7% African-American, 36.1% White; 3.9% Hispanic; 3.3% Other ethnicity	57.5 (9.4)	68	94	88	98
[34]	Bogner (2013)	Pilot Trial	USA	Community based primary care setting	Depression & Hypertension	Upcoming appointments records	In-person	12 weeks	0,6,12 weeks	50% White; 38.3% Black/African-American; 10% Hispanic/Spanish, 1.7% Asian/Pacific islander	67.1 (11)	65	30	30	100
[35]	Darby (2024)	Mixed methods study reporting on implementation and pre/post intervention measures	USA	Free clinic targeting an underserved population	Two or more chronic health problems	Primary care population of clinic	In- person, phone, online	48 months	0, 48 months	58% African American; 37% White; 5% Hispanic	42% aged ≥ 51 years	63	19	0	95
[36]	Dwight-Johnson (2005)	Randomised pilot study	USA	Public sector oncology clinics serving low-income Latino patients	Depression & Cancer	Clinical records	In-person, Phone	8 weeks^$^	0,4,8 months	100% Latina	47.1 (11.3)	100	28	27	N/A*
[37]	Ell (2008)	RCT	USA	Public sector oncology clinics serving low-income Latino patients	Depression & Cancer	Clinical charts	In-person, Phone	12 months	0,6,12 months	87.9% Hispanic	49% aged ≥ 50 years	85	242	230	55
[38]	Ell (2010)	RCT	USA	Public community clinics	Depression & Diabetes	Medical charts	In-person Phone	12 months	0,6,12,18 months	96.5% Hispanic	72% aged ≥ 50 years	82	193	194	71
[39]	Fedder (2003)	Retrospective comparison study(Pre-test/Post-test)	USA	Community setting	Diabetes & Hypertension	Discharge rolls	In-person, Phone	12 months	12 months pre & post intervention	100% African-American	57.4 (12)	78	117	0	49% had 5 + encounters with CHW & were included in intervention cohort
[40]	Gallo, (2023)	Randomised controlled single-blind parallel group study	USA	Community setting	Chronic cardiometabolic conditions (Obesity, Diabetes, Hypertension, Dyslipidaemia, Cerebrovascular or Cardiovascular diseases) & at least one behavioural health concern (e.g., Depression symptoms, Alcohol mis- use)	Electronic health records	In-person, Phone	30 days	0, 1, 3, 6 months	100% Hispanic/Latinos	62.3 (13.2)	48	271	265	67
[41]	McClintock (2017)	Pilot RCT	USA	Primary Care practices	Hypertension & Depression	Electronic health records and upcoming appointment records	In-person, Phone	12 weeks	0, 6,12, weeks	66% Black; 24% White; 3% Asian; 7% Other	62.3, (8)	74	29	25	98
[42]	O'Malley (2022)	Pilot RCT	USA	Ambulatory care settings	At least 3 comorbid medical conditions (one of which was Hypertension)	Patients visiting primary care	In person	1 visit	0,4,12 weeks	57% Black; 36% White; 7% Hispanic/Other	66	53	51	69	88
[43]	Taufique (2023)	Case study (Pre-test/Post-test)	USA	Specialised School Setting	Depression, anxiety, self-harm behaviours, restricted eating, social isolation	collaboration with education-academic-health system partnership	In-person	~ 9–12 months	0,3,6,9,12 months	100% Hispanic	15	100	1	0	100
[44]	Tobe (2006)	RCT	Canada	Community setting	Diabetes & Hypertension	community screening clinics and during home care visits	In person	12 months	0,1.5, 3,6,9,12 months	100% First Nations people	55.6, (12.1)	62	50	49	96
[45]	Zimbudzi (2023)	Longitudinal study (Pre-test/Post-test)	Australia	Tertiary hospital	Diabetes & Chronic Kidney Disease	Patients attending tertiary hospital	In person	12 months	0, 12 months	26% CALD; 75% non-CALD	67 (12)	36	77	213	57

^$^unclear so this figure is based on the number of sessions reported, *IntG* intervention group; *ComG* comparison group; *SD* standard deviation; *CALD* Culturally and linguistically diverse patients

^*^Not reported but authors indicate that dropout rates did not significantly differ between the collaborative intervention and usual care groups

#### Participant recruitment, participant characteristics, health conditions

In most studies, participants were recruited from existing patient populations [35] and were identified through clinical records, discharge rolls and upcoming patient appointments [31–34, 39–42, 45]. In three studies, bilingual study recruiters were trained to identify eligible patients from clinical charts [36–38]. In one study eligible participants were identified through community screening clinics and visits by homecare nurses and health aides [44]. One study did not explicitly outline their recruitment strategy but reported that their collaboration with, and support from, the New York City Department of Education allowed them unprecedented access to a large, representative adolescent population [43].

The number of participants included in the interventions varied, with the largest trial (randomised or otherwise) including 271 participants in the intervention group and 265 participants in the comparison group [40] (Table 1). The sample sizes across the four pre/post-test studies ranged from one participant, chosen as a case study from a larger cohort of participants [43] to 290 participants [45]. Reports of the number of participants with complete data at baseline and in the final assessment was reported in 14 studies. We used this data to calculate the retention rates which were generally high; eight studies had retention rates of 95% and above [31–35, 41, 43, 44]. Except for two studies where men were the majority population in the sample [40, 45], all other studies had a larger proportion of women, ranging from 53% [42] to 100% [36, 43]. Apart from one study which sought to improve the health outcomes of a 15-year-old girl with co-existing mental health and substance misuse disorders [43], all other studies recruited middle-aged or older participants. In three out of 15 studies, most participants were younger than 51 years or had a mean age below 50 years [35–37]. One study reported that 75% of their participants were above the age of 50 years [38]. In five studies most of the participants were in their 50s [31–33, 39, 44]. In five other studies most participants were in their 60s [34, 40–42, 45].

Of the 15 included studies, six studies recruited participants from a single minoritised racial group i.e. Hispanic/Latina/Latino (*n* = 3) [36, 40, 43], African American (*n* = 2) [32, 39], and First Nations people in Canada (*n* = 1) [44]. Hispanic people were the majority in two studies (i.e. 88% [37] and 97% [38]) and African Americans were the majority in five other studies (i.e. 81% [31], 66% [41], 57% [33], 58% [35] and 57% [42]. In one study, 50% of the participants were reported to be Black/African-American, Hispanic/Spanish or Asian/Pacific Islander [34]. Another study recruited people living in Australia with culturally and linguistically diverse backgrounds and/or were born in non-English speaking countries [45].

Twelve studies examined comorbidity with Depression, Hypertension, and Diabetes, being the most frequently cited index conditions (i.e. Depression and Hypertension [31, 34, 41], Depression and Diabetes [32, 33, 38], Depression and Cancer [36, 37], Diabetes and Hypertension [39, 44], Diabetes and Chronic Kidney Disease [45], and Hypertension and other comorbid conditions [42] (Table 2). In the remaining three studies, people were eligible to participate if they had ≥ 2 chronic health problems [35], > 2 long-term mental health conditions (i.e. Depression, Anxiety, Self-harm behaviours, Restricted eating, Social isolation [43]) or chronic cardiometabolic conditions (Obesity, Diabetes, Hypertension, Dyslipidaemia, Cerebrovascular or Cardiovascular diseases); and behavioural health concerns (e.g. Depression or Anxiety symptoms, disease-related distress, Chronic stress, Smoking, Alcohol misuse) [40]).

**Table 2:** Intervention characteristics, outcomes measure and results

Intervention details	Socio-cultural considerations	Outcome(s) of interest	Statistically significant results
Study ID [31]: Bogner (2008) Name: Integrated care intervention Theoretical underpinning: Integrated care informed by an adaptation of the theory of reasoned action^	Provision of an individualised program that recognises patients’ social and cultural context; Family, friends, community, stigma, cost of medication factored into the conceptual model	Depressive symptoms; Blood Pressure; Adherence to medication	Intervention group (IG) vs Usual care group (UCG) at 6 weeks Depressive symptoms—CES-D, mean (SD): 9.9 (10.7) vs 19.3 (15.2) (*p* = 0.006) Systolic blood pressure, mean (SD) mm Hg: 127.3 (17.7) vs 141.3 (18.8) (*p* = 0.003) Diastolic blood pressure, mean (SD) mm Hg:75.8 (10.7) vs 85.0 (11.9) (*p* = 0.002) % with > 80% adherence to antidepressant: 71.9 vs 31.3 (*p* = 0.001) % with > 80% adherence to antihypertensive: 78.1 vs:31.3 (*p* <.0001)
Study ID [32]: Bogner (2010) Name: Integrated care intervention Theoretical underpinning: Integrated care informed by an adaptation of the theory of reasoned action^	Cultural competence training for staff; The integrated care manager worked individually with patients to address factors involved in adherence (e.g. social support and cost of medication) outlined in the conceptual framework	Blood glycaemic control; Depression; Adherence to oral hypoglycaemic & antidepressants	IG vs UCG at 6 weeks: % with > 80% adherence to an oral hypoglycaemic: 62.1 vs 24.1(*p* < 0.01) % with > 80% adherence to antidepressant: 62.1 vs 10.3 (*p* < 0.001) IG vs UCG at 12 weeks: A1C levels, mean (SD): 6.7(2.3) vs 7.9(2.6) (*p* = 0.019) Depression CES-D, mean (SD): 9.6(9.4) vs 16.6(14.5) (*p* = 0.035)
Study ID [33]: Bogner (2012) Name: Integrated care intervention for patients with type 2 Diabetes and Depression Theoretical underpinning: Integrated care informed by an adaptation of the theory of reasoned action^	Provision of an individualised program congruent with patients’ social & cultural context; The integrated care manager worked individually with patients to address factors involved in adherence (e.g. social support and cost of medication) outlined in the conceptual framework	Adherence to antidepressants & oral hypoglycaemic agents; HbA1c level of less than 7%; Depression remission	IG vs UCG at 12 weeks % with ≥ 80% adherence to an oral hypoglycaemic: IG vs UCG: 65 vs 32 (*p* < 0.001) % with ≥ 80% adherence to antidepressant: IG vs UCG: 61 vs 22 (*p* <.001) Glucose control: % achieved HbA1c < 7%: IG vs UCG: 60.9 vs 35.7 (*p* < 0.001) Depression: % achieved remission (PHQ-9 < 5): IG vs UCG: 58.7 vs 30.7 (*p* < 0.001)
Study ID: [34] Bogner (2013) Name: Licensed Practical Nurse Intervention for Hypertension and Depression Theoretical underpinning: Integrated care informed by an adaptation of the theory of reasoned action^	Family incorporated into the conceptual model; Participants encouraged to involve a family member to assist in opening medication bottles & in the development of cognitive aids; Past experiences, side effects of medication, cost of medication, and system-level factors considered as factors affecting non-adherence and suggestions to address them considered	Blood pressure control (systolic and diastolic); Depressive symptoms (PHQ-9)	IG vs UCG at 12 weeks Diastolic blood pressure, mean (SD) mmHg 74.2 (13.6) vs 82.0 (15.7) (*p* = 0.035) Depression—PHQ-9, mean (SD) 2.4 (3.2) vs 7.1 (5.5) (*p* < 0.001)
Study ID: [35] Darby (2024) Name: Intensive Primary Care (IPC) Nursing Theoretical underpinning: Intensive Primary Care model utilising 5As as an Organizing Framework	Use of Community Health Workers (CHW) with shared ethnic and socio-cultural background; Cost estimations for medication conducted; Time set aside to find access to speciality services for patients without health insurance	Patient Assessment of Chronic Illness Care [PACIC]−20); Functional, Communicative, Critical Thinking Health Literacy; Perceived Stress; Patient Activation; Perceived Self Efficacy for Chronic Disease; EuroQoL- 5 Dimension; Trust in Provider; Emotional Support; Patient Health Questionnaire-9; Blood pressure, BMI, A1C levels	Pretest vs post-test scores PACIC-20 mean score (SD:) 56.5 (27.2) vs 87.9 (17.10) T (18) Score: 4.8 (*p* < 0.001) Patient activation mean score (SD): 42.8 (6.6) vs 48.3 (3.9) T (18) Score: 3.5 (*p* = 0.002) Self-efficacy/chronic disease mean score (SD): 35.6 (11.5) vs 48.7 (9) T (18) Score: 4.2 (*p* < 0.001) Trust in provider mean score (SD): 46.5 (19.6) vs 21.4 (10.4) T (18) Score: 4.6 (*p* < 0.001)
Study ID: [36] Dwight-Johnson (2005) Name: Multifaceted Oncology Depression Program/collaborative Care Intervention Theoretical underpinning: The Chronic Care Model	Use of bilingual recruiters and assessors; Medication and problem-solving therapy costs covered by the study; Patients could include family members in their treatment if they preferred	Patient death; Adhered to Cancer treatment; Depression symptom improvement; ≥ 50% reduction in PHQ-9 score; Quality of life (i.e. physical, social/family, emotional, functional well-being)	Results at 8 months Likelihood Patient death in UCG relative to IG Odds Ratio (OR): 0.04, 95% Confidence Interval (CI) 0.002–0.74 X^2^ = 9.71, *p* = 0.002 Likelihood of Depression symptom improvement in IG relative to UCG OR: 3.33, 95% CI: 1.05–10.59, X^2^ = 4.32, *p* = 0.04 Likelihood of ≥ 50% reduction in PHQ-9 score in IG relative to UCG OR: 4.51 95% CI: 1.07–18.93, X^2^ = 4.65, *p* = 0.03 Emotional well-being in IG vs UCG: 2.15 (3.56) vs −0.50 (5.26) Group difference mean 2.65 (95% CI): (0.18 to 5.12), F statistic: 5.24 (*p* = 0.03)
Study ID: [37] Ell (2008) Name: Alleviating Depression Among Patients with Cancer (ADAPt-C) collaborative care management for major Depression or Dysthymia Theoretical underpinning: Impact stepped care model/collaborative care model	Use of bilingual recruiter; Brief cultural competency training for staff and intervention adaptations to enhance acceptability; Use of Spanish speaking research staff; Study and intervention materials translated to Spanish and adapted for literacy and idiomatic content; Telephone data collection and intervention option provided; Evening and weekend telephone visits and scheduling Depression treatment visits available to coincide with oncology appointments; Attention to family member roles; Reimbursement for time in completing outcome interviews and for transportation; Copays for antidepressant medication if indicated	Depression outcomes; Quality of life outcomes; Severity of Depression; Health-related quality of life: Physical, functional, social, and emotional well-being; Satisfaction with the treatment relationship; Pain, physical and mental health	Results at 12 months IG vs EUCG Likelihood of 50% PHQ-9 reduction IG relative to EUC OR: 1.98, 95% CI: 1.16 to 3.38 (*p* = 0.01) Likelihood of 5-pointPHQ-9 reduction in IG relative to EUC OR: 1.99, 95% CI: 1.14 to 3.50 (*p* = 0.02) Rates of Depression treatment: 72.3% v 10.4% (*p* < 0.0001) Social/family well-being^*^: 2.7 (1.22 to 4.17) *p* = 0.001 Emotional well-being^*^: 1.29 (0.26 to 2.22) *p* = 0.01 Functional well-being*: 1.34 (0.08 to 2.59) *p* = 0.04 SF-12physical^*^: 2.79 (0.49 to 5.1) *p* = 0.02 ^*^Adjusted mean difference (95% CI)
Study ID: [38] Ell (2010) Name: Multifaceted Diabetes and Depression Program Theoretical underpinning: Collaborative Care Model/evidence-based practice guidelines for primary care and are responsive to known barriers to treatment among patients in public safety-net clinics	Use of bilingual recruiters and cultural intervention adaptations e.g. psychoeducation to dispel treatment misconceptions, reduce stigma, and enhance therapeutic alliance; Patient choice of first-line medication or Problem Solving Therapy (PST); Family participation permitted; PST sessions tailored for literacy and idiomatic content and linking PST problem solving skills training to enhance diabetes and Depression self-management and coping with socioeconomic stress; Patient navigation assistance provided to facilitate patient-provider communication and financial and social resource access	Depressive symptom outcomes; Functioning, quality of life, anxiety, adherence, and A1C levels Secondary outcomes: self-reported weight and height (BMI), diabetes complications, comorbid medical illness, and socioeconomic stress (financial situation, work, unemployment, financial problems, marital/family conflicts, legal and care-giving problems, and community violence worry	Results at 18 months (IG vs EUC) % in receipt of antidepressant: 36.1 vs 19.7 (*p* = 0.002) % in receipt of PST/counselling: 24.3 vs 12.4 (*p* = 0.01) % in receipt of any Depression treatments: 45.8 vs 24.1 (*p* < 0.001) % Response (50%SCL-20 reduction): 61.8 vs 43.8 (*p* < 0.001) % Remission (SCL-20 0.5): 40.3 vs 35.0 (*p* = 0.04) Outcomes with significant time by group interactions, *p* < 0.001 Sheehan Disability Scale of functional impairment, pain impact, Mental Component Summary-12 scale, financial situation, Number of social stressors Outcomes with significant time by group interactions, *p* = 0.04 Whitty-9 diabetes symptoms, Physical Component Summary-12 scale
Study ID: [39] Fedder (2003) Name: Community Health Worker Outreach Program Theoretical underpinning: Indigenous health worker case manager model	Use of CHW with shared ethnic and socio-cultural background; assisting in establishing and/or sustaining Medicaid eligibility if appropriate; and providing social support to patients, their caregivers, and families	Emergency Room visits (all); Emergency Room admissions; Total hospital admissions; Length of hospital stay; Medicaid reimbursements, Quality of life	Within group change, Mean, (SE) [%] Emergency Room admissions: −0.32(0.1) [−53] (*p* = 0.02) Length of hospital stay: +0.34 (2.6) [+5] (*p* = 0.02) Medicaid reimbursements ($): −2246 (1792) [−27] (*p* = 0.01)
Study ID: [40] Gallo, (2023) Name: "Mi Puente (“My Bridge”; MP) Care transition program Theoretical underpinning: Ideal Transitions in Care (ITC) framework	Family involvement permitted; Mentors shared ethnic and socio-cultural background; At baseline, participants reported sociodemographic characteristics and their preferred language	*Primary outcome*: Hospital readmissions (inpatient, emergency department, observation) within 30- and 180-days post-discharge *Secondary outcomes:* Count of readmissions and total inpatient length of stay Patient-Reported Outcomes Measurement Information System Global-10 Health Scale, 13- item Patient Activation Measure and a short version of the Chronic Illness Resources Survey Patient self-report on inpatient days, healthcare use in the past 3 months, urgent care visits, and poor healthcare access	Intention-to-treat models, IG vs UCG at 30 days Rate of recurrent hospitalization: 15.9% vs 9.4% (OR = 1.91 (95% CI 1.09, 3.33)), Number of recurrent hospitalizations, mean (SD): 0.20 (0.49) vs 0.12 (0.45) *p* = 0.02)
Study ID: [41] McClintock (2017) Name: Integrated intervention for Hypertension and Depression incorporating patients’ social determinants of health Theoretical underpinning: A broader framework incorporating a social lens which addresses both biomedical needs and social determinants of health in Hypertension and Depression care	Use of Patient Prioritised Planning which recognises patients’ social and cultural context by addressing health-related priorities identified by patient	Depressive symptoms; Systolic blood pressure; Diastolic blood pressure	Enhanced intervention group (EIG) vs Basic intervention group (BIG) at 12 weeks Depression- PHQ-9, mean change from baseline (SD): − 2.75 (0.93) vs 0.40 (0.98) Between group difference (95% CI): 3.15 (0.44–5.86) *p* = 0.024 Systolic blood pressure, mean change from baseline (SD): − 11.96(3.35) vs 6.08(4.94) Between group difference (95% CI): 18 (6.28–29.81) *p* = 0.003 Diastolic blood pressure, mean change from baseline (SD): − 4.79 (1.69) vs 4.12 (3.39) Between group difference (95% CI): 8.91 (1.05–16.26) *p* = 0.019
Study ID: [42] O'Malley (2022) Name: Pre-visit Patient Activation Card Theoretical underpinning: Not Stated	Patient activation card co-developed with patients and physicians	*Primary outcome*: Change in medication adherence as measured by a pill count at 4 and 12 weeks; *Secondary outcomes*: Patient satisfaction; Trust in one’s physician; Physician rating of the encounter as “difficult” Patient activation; Functional status and change in blood pressure at 4 and 12 weeks	Post visit Patient activation, Mean score (SD): IG vs UCG 4.4 (1.57) vs 3.8 (1.53) *p* = 0.047
Study ID: [43] Taufique (2023) Name: Comprehensive Adolescent Rehabilitation and Education Service (CARES) Theoretical underpinning: Group, Transtheoretical Model for Change	Program commitments: (1) recovery-oriented care that is consumer driven, based on hope and delivered with dignity, that recognizes the critical role of family and other relationships in a person’s life, and recognizes that the ability to be gainfully employed and contribute to one’s community are essential to quality of life and to self-regard. (2) culturally and linguistically competent care Family involvement permitted: Family Therapy (1x/week); Crisis Management (as needed) with patient or with family	Substance use; Mental Health; Aggressive Behaviours; Physical health; Family, Peer relations; Social Skills; Leisure/Recreation; Educational; Vocational	Results at 12 months: Pre-test vs Post-test^$^ Substance Use: 6/17 vs 6/17; (URICA: 6.4,pre vs 13, action) Mental Health: 13/22 vs 7/22; (URICA: no problems identified vs 12.7, action) Aggressive Behaviours: 8/16 vs 4/16 Physical Health: 5/10 vs 3/10 Family: 5/11 vs 1/11 Peer relations: 7/10 vs 2/10 Social Skills: 7/11 vs 3/11 Education: 10/26 vs 9/26
Study ID: [44] Tobe (2006) Name: Nurse-Directed Hypertension treatment Theoretical underpinning: Home and community model	Participants accompanied by home health aide to help with linguistic and cultural issues	*Primary outcome*: Change in systolic blood pressure after 12 months *Secondary outcomes*: Change in diastolic blood pressure over time; Change in urine albumin status; Incidence of adverse events	Change over time from baseline to final visit, IG vs Control group (CG) Diastolic blood pressure, mmHg, Mean (SD): –11.6 (10.6)* vs –6.8 (11.1)* *p* = 0.05 for comparison between groups over time *p < 0.001 for comparison within group over time
Study ID: [45] Zimbudzi (2023) Name: Codesigned integrated kidney and diabetes model of care Theoretical underpinning: Person-centred integrated models of care	Model of care co-developed with stakeholders; Utilisation of professional interpreting services in preference to family members for non‐English speaking patients	Patient activation scores	Within group change from base line Patient Activation Scores: CALD background: 7 points (52.1 ± 17.6 to 59.4 ± 14.7) [significant changes] non-CALD background: 0.3 point (58.5 ± 14.6 to 58.8 ± 13.6) [non-significant changes] Baseline differences in patient activation scores between groups were significant (*p* = 0.002) but differences in patient activation scores at 12 months were non-significant (*p* = 0.71)

^adapted from Cooper et al. [46] ;^$^*p*-values not reported; *BMI *Body Mass Index; *CALD* Culturally and linguistically diverse; *PST* Problem Solving Therapy; *URICA* University of Rhode Island Change Assessment Questionnaire

**Fig. 3:**
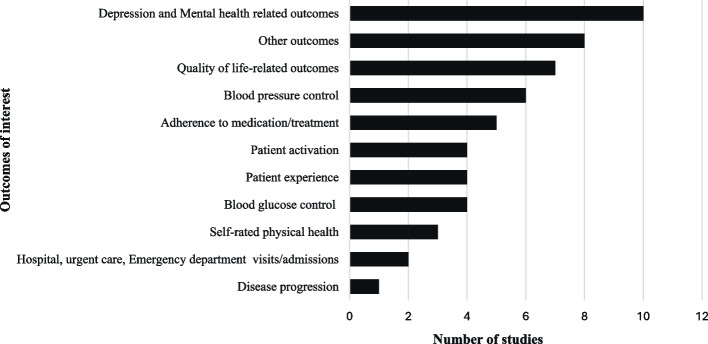
Outcomes measured in included studies with the number of studies per outcome

We present the intervention types, theoretical underpinning, approaches to care, outcomes and results in Table 2. Fig. 3 below lists the outcomes extracted from the 15 included studies. Depression and mental health related outcomes (*n* = 10), Quality of Life/Quality of Life-related measures (*n* = 8), blood pressure control (*n* = 6), and adherence to medication (*n* = 6), were the most frequently assessed outcomes. Other outcomes included substance use, aggressive behaviours[43], self-management resources [40], emotional support, perceived self-efficacy for chronic disease [35], Body Mass Index [35, 38], socioeconomic stress [38], mortality [36], incidence of adverse events, urine albumin status change [44], Medicaid reimbursements [39], and physician-related outcomes [42]. All studies focused on patient-related outcomes, but one study assessed healthcare staff-related outcomes i.e. physician ratings of encounters with patients [42]. Two studies considered outcomes linked to the social determinants of health educational outcomes, vocational outcomes [43], unemployment, marital/family conflicts, legal and care-giving problems, community violence worry and financial situation/problems [38].

#### Intervention types, theoretical underpinnings, and approaches to care

Of the 15 interventions, six were integrated care interventions where care managers/interventionists/clinical staff coordinated care, supported patients to manage their conditions and acted as liaisons between patients, other healthcare staff and educational staff [31–33, 41, 43, 45] (Table 2). Three interventions were collaborative care interventions which adopted multifaceted approaches to care delivered by multidisciplinary teams (e.g. problem-solving therapy, motivation, coping strategies, systems navigation, symptom monitoring, support with medication adherence, use of structured algorithms for stepped care management) [36–38]. Three interventions were nurse-directed and involved nurses following a pre-defined treatment algorithm [44], leading care with the assistance of community health workers (CHWs) [35] and coordinating care in collaboration with physicians [34]. In one intervention, participants received support from a team consisting of a nurse, who provided medical support, and a community mentor, who leveraged community resources to support patients with social determinants of health [40]. Another intervention was led by CHWs supporting participants with health service navigation, linkage with healthcare staff, and symptom management [39]. In the last intervention, a Patient Activation Card was used to prompt patients to reflect on their goals for their appointment, prioritise those goals and discuss their concerns with their physicians [42].

Except for one study, [42], all interventions were underpinned by or evaluated using a theory/model/framework. Four interventions [31–34] were informed by an adapted theory of reasoned action which posits that a patient’s behavioural intention and eventual treatment-seeking is determined by their evaluation of the potential risks and benefits of that behaviour [46]. Internal factors (e.g. attitudes, beliefs, and social norms) and external factors (e.g. demographics, severity of condition, social support) influence behaviour. While internal factors are modifiable through education or experience, external factors are modifiable only with substantial effort if at all [46]. In these studies, patients worked with healthcare providers to identify and address barriers to medication adherence and optimal care.

Other studies employed similar models which also encourage collaborative working across the stakeholders in the health system and community. These include the Intensive Primary Care model [35], the Person-centred Integrated Model of Care [45], Chronic Care Model [36], the Collaborative Care Model [37, 38], Group-As-A-Whole, and Transtheoretical Model for Change [43]. In these studies, trained social workers, GPs, nurses/specialists worked with other professionals to deliver care and improve patient outcomes. In one of these interventions, community Cancer resources were employed to support patient needs [36].

Some interventions were underpinned by bio-psycho-social frameworks and blended different approaches to care e.g., a framework incorporating Patient Prioritised Planning with a social lens addressing biomedical needs and social determinants in health [41], Home and Community Model [44], and the Indigenous Health Worker Case Manager model [39]. These interventions also emphasised the importance of community settings [44], and/or CHWs [39] and the need to address contextual factors (e.g. housing, transport, emotional, and financial needs which are key to understanding the challenges hindering optimal disease management) [41]. The Intensive Primary Care Nursing intervention which focused on supporting the full use of a nurse’s experience/training was underpinned by the classic 5As framework (Assessment, Advise, Agree, Assist, and Arrange) and centred patients’ needs [35]. However, it also adopted a community-based perspective where nurses worked with CHWs in a free clinic with a longstanding history of community outreach, providing screening services, and health education to patients in non-clinical settings [35].

The focus of one intervention was on systems for delivering and monitoring treatment and was underpinned by the Collaborative Care Model as well as the Impacted Stepped Care Model [37]. Another intervention, which focused on patient transitions, was underpinned by the Ideal Transitions in Care framework and leveraged community resources to address adverse social determinants and other barriers and improve the outcomes of people with MLTCs [40]. The one study with no underlying framework incorporated changes to the patient pathways by introducing a Patient Activation Card to improve patient-provider communication [42].

#### Adaptations/enhancements to promote equitable and inclusive care

All studies recognised the social, cultural and racial/ethnic diversity of the patient population and enhanced their interventions to provide equitable and inclusive care in different ways. Some interventions adopted individualised approaches to care which recognise patients’ social and cultural context by addressing health-related priorities identified by the patient [31, 33, 41]. Others provided linguistically competent care by ensuring that participants with language difficulties were assisted by interpreters who were available at all stages of the study [37, 38, 40, 44, 45]. In two studies, intervention materials were not only translated but also adapted for literacy and idiomatic content [37, 38]. In three studies, bilingual recruiters assisted with the identification of eligible participants from clinical records [36–38].

Elsewhere, CHWs and mentors who facilitated the intervention shared the same racial, and cultural background with the participants and in some cases, lived in the same neighbourhoods and experienced the same health conditions [35, 39, 40]. Using facilitators with shared characteristics was believed to promote trust and understanding between patients and providers. Facilitators who had shared health experiences, were seen as ideally suited to communicate empathetically with patients having had similar personal experiences and concerns [39].

Seven interventions recognised the role of family members in the participants’ life [31, 34, 36–38, 40, 43] and involvement of family members in some interventions was encouraged/permitted [34, 36–38, 40, 43]. In one study, the decision to involve family members was based on evidence that family participation in healthcare provision is important in Hispanic/Latino culture [40].

Two interventions reported the involvement of stakeholders in the development of the intervention [42, 45]. The Patient Activation Card used in the intervention to improve patient-provider interactions in adult primary care was developed using a series of focus groups of physicians and patients [42]. Similarly, the model of care adopted in the Integrated Kidney and Diabetes intervention was codesigned by patients, caregivers and national consumer advocacy organisations working with healthcare professionals and researchers [45]. The patients and caregivers also provided a robust evaluation of the co-developed model of care [45].

Cultural competence training was described in two studies [32, 37]. For example, in the collaborative care intervention for low-income Cancer patients with Diabetes, staff received a brief and self-administered training in cultural competence [37]. In contrast, in the intervention which integrated Diabetes and Depression care among African Americans, the integrated care manager received training delivered during weekly sessions by the principal investigator [32]. The training included cultural interviewing and sensitivity training as well as culturally competent evidence-based practices to quickly build rapport within primary care settings [32]. In this study, the integrated care manager conducted cultural interviewing to help participants devise culturally acceptable solutions to nonadherence and diabetes information was tailored to their cultural context [32].

#### Social factors which influence health

In addition to cultural and linguistic considerations, social factors which are known to influence healthcare were acknowledged and addressed [35–38, 40]. In some interventions, participants were supported with access to resources, and reimbursed for time, transportation, and medication [37, 38]. They were also provided with choice in treatment, appointment time, and mode of delivery [37, 38].

Community mentors in the Mi Puente Care Transition Program, provided support and leveraged community resources to address adverse social determinants and other barriers [40]. Similarly, in the Multifaceted Oncology Depression Program, the Cancer/Depression specialist used community Cancer resources to assist patients with transportation, social support, and education [36]. The cost of medication was also considered in some interventions [31, 34–36]. For example, in the Multifaceted Oncology Depression Program, medication and problem-solving therapy costs were covered by the study [36]. Similarly, in the Intensive Primary Care Nursing intervention for patients with complex medical and social needs, facilitators used cost estimating online applications for medication crucial for patients’ treatment and set aside time to identify access to specialty services for those without health insurance [35].

### Impact of intervention on outcomes of interest

Of the 15 included studies, five studies led to statistically significant improvements in all outcome measures for participants in the intervention group compared to the comparison group at final follow-up [31–33, 41, 45]. Nine interventions led to statistically significant improvements in some, but not all, outcomes measures [34–39, 42–44]. Only one intervention resulted in better outcomes for the usual care group when compared to the intervention group at final follow-up [40]. A list of the statistically significant results (*p* < 0.05) is presented in Table 2.

Collectively, the interventions resulted in statistically significant improvements in a) clinical outcomes (e.g. diabetes symptoms [38], blood glucose control [33, 34], diastolic blood pressure [31, 34, 41, 44], systolic blood pressure [31, 41], patient death [36]), b) mental health outcomes (i.e. Depression, depressive symptoms [31–34, 36–38], mental health scores [38, 43]), c) health service utilisation outcomes, (e.g. length of hospital stay, ER admissions [39], d) health-related patient behaviours (i.e. patient activation [35, 42, 45] d) treatment, medication use and adherence outcomes (i.e. adherence to Diabetes medication [32–34], Hypertension medication [31], Depression medication/treatment [31–34, 37, 38] and e) patient-reported outcome measures (including socioeconomic outcomes) (e.g. trust in provider, perceived self-efficacy for chronic disease [35], emotional wellbeing [36, 37], physical health [37, 38, 43], functional wellbeing [37, 38], chronic illness care [35], substance use, social skills, aggressive behaviour, education [43], pain impact [38], family, peer, social wellbeing [38, 43] number of stressors, financial situation [38], and Medicaid reimbursements [39].

### Quality of evidence

The quality assessment of the four pre-test/post-test studies revealed no critical issues with the study design and execution (Supplementary Material 3). All authors specified and clearly defined their aims, objectives, and outcome measures [35, 39, 43, 45]. However, with exception of Darby, Smith [35] who explicitly reported that the analysis was conducted by a statistician not involved in the intervention, there was a lack of information regarding blinding of the personnel in the other studies [39, 43, 45]. Selective reporting was considered to have occurred in the Community Health Worker outreach program due to the lack of evidence supporting improvements in quality-of-life outcomes [39]. Similarly, confidence in the study results of the CARES intervention was reduced because of the small sample size (*n* = 1) and the lack of *p-*values when reporting the results [43].

**Fig. 4:**
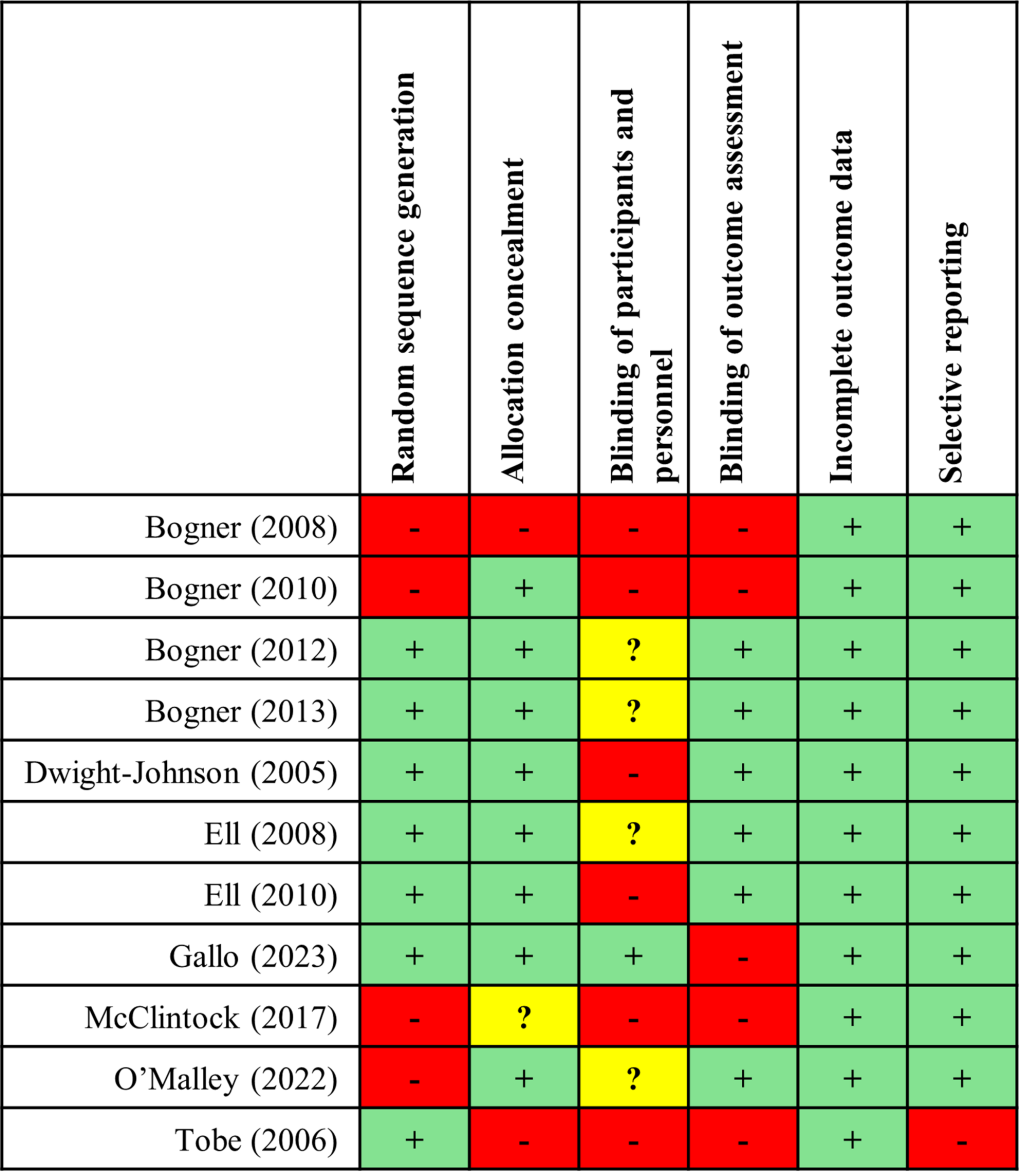
Risk of bias summary based on review authors' judgements

**Fig. 5:**
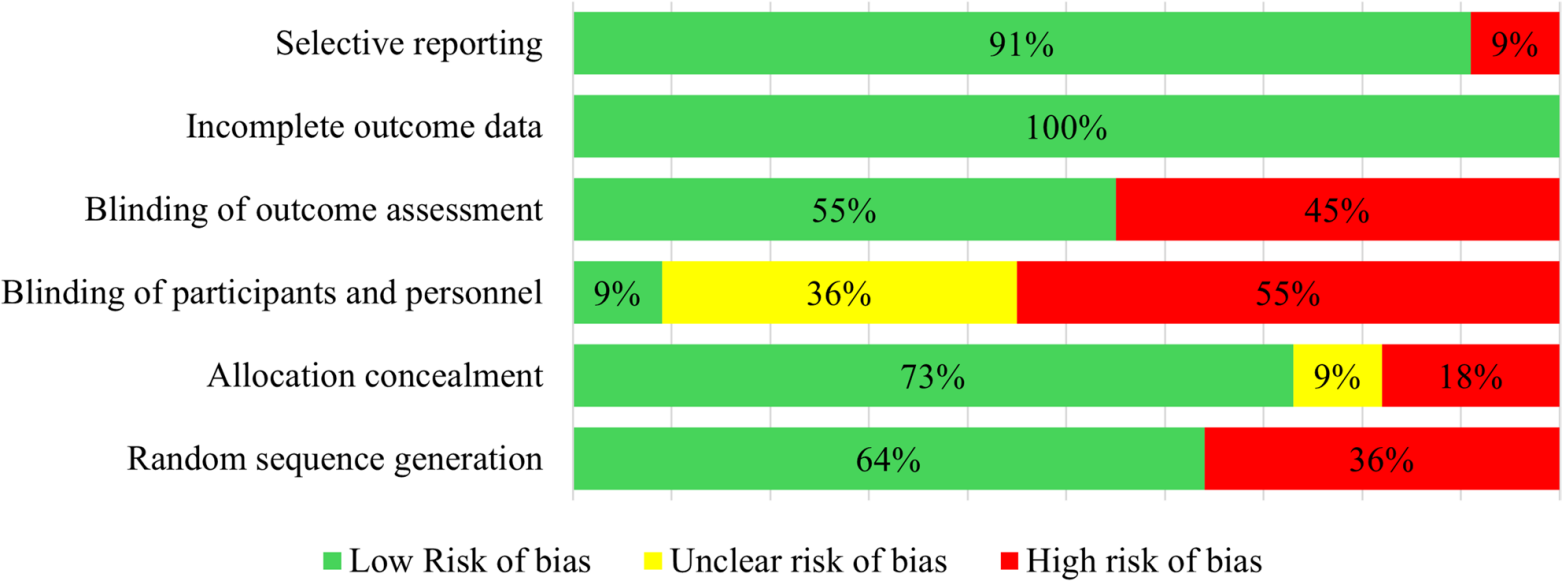
Risk of bias graph based on authors’ assessment

The summary assessment of the risk of bias for the included trials is provided in Figs. 4 and 5. Random sequence generation was considered not to have been conducted in four studies [31, 32, 41, 42]. Allocation concealment was deemed satisfactory in eight studies but was considered unclear in one study [42] and inadequate in two other studies [44]. Blinding of participants/personnel was judged to be adequate in one study only [40]. Similarly, blinding of the outcome assessment was considered adequate in six studies [33, 34, 36, 37, 42]. In one study, the authors noted that blinding of the study was not done as it was unacceptable to conceal treatment among First Nations people with existing Hypertension and Diabetes [44]. Risk of bias arising from incomplete reporting of outcome data was assessed to be low in all studies and selective reporting was considered to have occurred in one study [44].

## Discussion

### Summary of findings

This review set out to systematically identify, describe and synthesise evidence from healthcare interventions aimed at improving healthcare outcomes for racially/ethnic minoritised people with MLTCs. 15 interventions met our inclusion criteria, 13 of which were conducted in the US. Most studies focused on comorbidity, with Depression being the most cited index condition. Depression symptoms, mental health, quality of life, blood pressure control and, adherence to treatment were the most frequently assessed outcome measures. Most interventions adopted an integrated, individualised approach to care administered through multidisciplinary health and care staff working collaboratively. Whilst the focus of some interventions was on identifying barriers to treatment and adherence to medication, most studies employed theoretical frameworks which recognised the influence of social determinants of health and importance of community resources (including CHWs, and family members) for improving the health and wellbeing of racially/ethnic minoritised people with MLTCs. A handful of studies were nurse-led, showcasing the possibility of training existing staff to take on clinical duties that have the potential to enhance and expedite care and treatment for people with MLTCs through continuity of care, care coordination and personalised care.

These interventions address processes at the micro-level and meso-level in Fig. 1 (e.g. nonadherence, unhelpful coping mechanisms, lack of interpreters, fragmented care, and lack of support). Examples of intervention components that address cultural preferences include the involvement of family members, using bilingual facilitators [36], and translating intervention materials [37]. The interventions also address some processes at the macro-level (e.g. low income, inequalities in education, neighbourhood deprivation, under-resourced practices, and socio-cultural processes). Examples of intervention components that address these structural barriers include reimbursement of travel and medication costs, assisting participants with no health insurance [35], and leveraging community assets to address adverse social determinants of health [40]. It is important to note that some interventions components addressed both structural barriers and cultural preferences, thereby cutting across all levels in Fig. 1. For example, co-producing interventions [42, 45] with racially minoritised people allows them to be part of the decision-making process and can highlight socio-cultural issues, barriers to services and increase uptake of services. Similarly, use of bilingual facilitators, translation of materials [36, 37], cultural competence training [32, 37] and the use of community health workers with shared racial/cultural backgrounds and experiences [35, 39, 40] can address misunderstanding of health conditions (micro-level) and issues of mistrust, cultural insensitivity, stereotyping and bias (macro-level) (Fig. 1).

Collectively, the interventions led to improvements in clinical outcomes, mental health, service utilisation, health-related patient behaviour, medication use and adherence, patient-reported outcome measures and selected socioeconomic outcomes. Our review provides evidence that racially/ethnic minoritised people can be recruited as participants in high quality clinical trials/interventions and that their health outcomes can be improved.

### Comparison with the literature

To our knowledge, this is the first systematic review that has set out to identify and synthesise evidence from interventions targeted at healthcare providers which seek to improve health outcomes for racially/ethnic minoritised people with multiple long-term conditions. A scoping review which seeks to identify and classify health promotion interventions targeted at racially/ethnic minoritised people with MLTCs is underway [47]. The scoping review seeks to identify all types of health promotion interventions at the individual, community, clinical and policy-level implemented to improve healthcare outcomes [47]. Our aims differ from the scoping review as we home in on health interventions targeted at healthcare providers/settings. In doing so, we provide a nuanced understanding of which healthcare interventions exist, their characteristics and their impact on outcomes of interest. We acknowledge the importance of identifying and understanding the mechanisms and processes attributed to (un)successful interventions and provide an in-depth account of these processes elsewhere [48].

Despite focusing on healthcare interventions, we identified the important role of community and its resources and the potential role of family members in improving health outcomes for racially/ethnic minoritised people. Our findings are supported by that of a review of behaviour change interventions for Pacific people which highlighted the importance of community-created, community-owned, culturally anchored, and collective approaches [49]. Although, the types of interventions examined were different, both reviews identified the importance of community and its assets. Our findings illustrate that community stakeholders are integral to the health system and not separate from it, and sustainable improvements in health inequalities can only be achieved through meaningful partnership between healthcare providers, and with community members and organisations [50].

It is well established that cultural competence training can equip healthcare professionals with the skills and knowledge required to provide equitable and inclusive care [51]. In this review, two studies provided details of a comprehensive cultural competence training for the facilitators [32, 38]. The lack of cultural competence training for facilitators delivering interventions aimed at racially/ethnic minoritised people might be deemed problematic particularly because such training can be used to combat racism, discrimination, cultural stereotyping, prejudice, and bias at the macro-level (Fig. 1). However, socio-cultural adaptations were made in several intervention to assure equitable and inclusive care. For example, some studies used bilingual staff/material to address language barriers, co-developed the intervention with stakeholders to assure suitability of the intervention, and involved family members (if preferred) in recognition of their importance in some cultures. Other studies used facilitators and CHWs who shared a similar background to patients (i.e. racial identity, language, socioeconomic status, lived experience of health conditions) to promote recruitment, build trust, and enhance engagement. The use of CHWs is a commonly used approach to assure cultural competency [52] and, therefore, address racism, discrimination and socio-cultural processes at the macro-level (Fig. 1). In fact, Fedder, Chang [39] argue that the success of their intervention, which involved CHWs with shared characteristics, suggests that *intervention materials may not need to be ‘‘culturally sensitive’’; i.e., specially prepared for the audience, as long as the interveners are* (p.26). Future research is required to identify which adaptations are most appropriate, in which circumstances, and the extent to which they are acceptable to racially/ethnic minoritised people and effective in improving their health outcomes.

In our review, Depression was the most frequently cited index condition alongside Hypertension, Diabetes, Cancer, and other mental health conditions. The extant literature suggests that racially/ethnic minoritised people are less likely to access timely, culturally sensitive mental health support in primary care [53]. For those who have accessed support services, the care provided has often been described as inadequate to meet their needs. The absence of socio-cultural considerations often contributes to poor care experiences [53]. Our review provides evidence of the feasibility of interventions that address both the mental and physical health of racially/ethnic minoritised people with MLTCs in one setting. Given that four of these interventions resulted in improvements in all mental and physical health outcomes of interest [31–33, 41], our review provides evidence of the feasibility and effectiveness of the integration of mental/physical healthcare. These interventions serve as an exemplar of how a single program in primary care can be used to address and improve the mental and physical health of racially/ethnic minoritised people with MLTCs.

### Areas for future research

Our review highlights a dearth of literature focused on racially/ethnic minoritised people who do not live in the US. Racially/ethnic minoritised people are a heterogenous population with varied migration patterns, languages and age profiles, living in different countries whose health and social care systems differ markedly. Thus, these findings cannot be generalised to other minoritised people living beyond North America and Australia. Future research should carefully consider the generalisability of these findings and the adaptations that may be needed before implementing similar interventions in other countries with a high proportion of racially/ethnic minoritised people with different health systems. There is an urgent need to address the lack of interventions for racially/ethnic minoritised people living in European countries, some of which have sizeable numbers of racially/ethnic minoritised people with MLTCs [54].

Women and older people were overrepresented in the interventions which were included in this review. This is unsurprising given that research shows that the prevalence of MLTCs increases with age and is higher among women than men [17]. It is important to note that the prevalence of MLTCs varies when different conditions are considered. Therefore, more research is needed to identify and assess whether particular clusters of long-term conditions are more likely to affect men or younger populations and identify how best to address them.

Disease control, quality of life and function are considered key outcomes of interest in interventions targeted at people with MLTCs [55]. In this review, Depression/mental health outcomes, patient-reported outcomes (particularly, quality of life and related measures), clinical outcomes, medication use, and adherence were the most frequently cited outcomes of interest. Only one study included provider-related outcomes [42]. In this study, a co-developed Pre-visit Patient Activation Card was used to prompted participants to reflect on their goals and expectations for the visit, and to define a prioritised agenda [42]. Alongside the patient-related outcomes, physician’s perceived difficulty of the encounter with patients was assessed [42]. The intervention did not lead to statistically significant improvements in this provider-related outcome. Given our focus on interventions targeted at healthcare professionals/systems/organisations, the paucity of studies reporting on provider-related outcomes is surprising and signals a priority research gap. Understanding the ways in which interventions impact health providers and delivery of care is crucial for improving sustainable, care quality which, in turn, benefits patients. This is particularly pertinent for racially/ethnic minoritised people, many of whom have a historic mistrust of healthcare providers and have faced discrimination in healthcare settings [56]. Future research should explore and identify provider-related outcomes of interest with the most potential to improve care delivery patients with MLTCs. These outcomes could include provider burnout/burden, staff skill mix, workforce retention, interprofessional collaboration/communication, or perceived cultural safety.

Except for three studies that used predefined algorithms to ensure that patients received care/treatment consistent with their clinical presentations and preferences [37, 38, 44], there were no studies which implemented digital interventions to improve health outcomes for racially/ethnic minoritised people with MLTCs. Digital interventions have the potential to enhance healthcare access, increase efficiency, address healthcare staff shortages, lower operational costs, [57, 58]. Findings from a recent systematic review suggests that culturally adapted digital mental health interventions are both effective and acceptable for racially/ethnic minoritised people [59]. Such interventions can circumvent barriers to mental health treatment and improve mental health equity among racially/ethnic minoritised communities. However, concerns have been raised about the potential for digital approaches to reproduce existing structural bias and inequity resulting in further marginalisation for vulnerable populations [57, 58]. Future studies are required to assess the feasibility, efficacy and acceptability of digital interventions for improving health outcomes for racially/ethnic minoritised people with MLTCs. To ensure they are equitable, digital interventions would need to be coproduced with key stakeholders (e.g. racially/ethnic minoritised people with MLTCs, their carers, and healthcare providers), adapted to local norms, and designed for both access and usability using representative data in training and testing.

### Strengths and limitations

This review has several strengths. First, we registered the protocol on PROSPERO [25] and used the PRISMA checklist [60] to report the findings and assure the quality of this review (Supplementary Material 5). We also refreshed the search in August 2024 and 2025 to ensure that the most recent studies were included in the review. Our review highlights the state of the art in healthcare interventions for racially/ethnic minoritised people with MLTCs and identifies gaps to be addressed by future research. The review has showcased the range of interventions that have been successfully implemented to improve health outcomes for racially/ethnic minoritised people with MLTCs.

Despite these strengths, our review has its limitations. For example, the blanket use of the term ‘multiple long-term conditions’, ‘multimorbidity’, ‘co-morbidity’ and their associated synonyms in the search terms may have inadvertently excluded studies that may have not used these terms but used the health conditions of interest (e.g. Diabetes, Hypertension). However, most of the studies we identified were studies of comorbidity where authors specified an index condition. Secondly, due to limited resources, approximately 90% of the studies were assigned to a single reviewer for single screening, data extraction and quality assessment. Thus, there may have been a high risk of reviewer bias during these stages of the review and key studies may have been missed. However, screening, data extraction and quality assessment were conducted in batches. Thus, before a reviewer started screening their allocated batch of studies, 10% of the studies were double screened with BH and discrepancies were discussed to reduce reviewer bias in these processes. Relatedly, the identification of themes was conducted by one reviewer (BH) who then discussed the themes with PO and MK. These themes were then written up and presented to the rest of the review team for commentary and feedback.

## Conclusion

Addressing the rising MLTCs challenge facing individuals and health systems is a significant public health priority for many countries [1]. Whilst our review illuminates research gaps that need to be addressed to inform strategies that can redress deeply entrenched racial/ethnic inequalities in healthcare, it illustrates the feasibility of implementing effective, socio-culturally sensitive interventions, many of which successfully integrate physical and mental healthcare, to significantly improve health outcomes for racially/ethnic minoritised people with MLTCs. Given that the prevalence of MLTCs and the health impact, care quality and experience of primary care for people with MLTCs varies markedly across racial/ethnic groups, it is vital to generate localised evidence around effective interventions that will improve healthcare outcomes for different racially/ethnic minoritised people with MLTCs in different contexts.

## Supplementary Information

Supplementary Material 1.

Supplementary Material 2.

Supplementary Material 3.

Supplementary Material 4.

Supplementary Material 5.

## Data Availability

All data synthesised in this review are available in the public domain.

## Footnotes

1 In this paper, we use the term ‘racially/ethnic minoritised’ to capture the different labels used to refer to people who do not self-identify as belonging to the white majority ethnic group in countries such as the UK and the US. The term ‘minoritised’ is used to emphasise how social positions are social constructions [61]. It also refers to people with shared experiences of exposure to interpersonal, institutional and structural racism [62] and discourages the literal reading of the minority/majority dichotomy which can reduce the issue of power relations to one of numbers [63].

2 3-D jobs (dirty, dangerous and demanding/demeaning) are low paying, labour intensive, precarious, hazardous, often invisible from the public orpublic policy and increase the risk of injury, and poor mental/ physical health [64]

3 In February 2023, we identified a database of interventions for long-term conditions on the Advancing Health Equity website. The website has since had an update, and the database is no longer available.
