# Supplementary material for: Healthcare interventions to improve health outcomes for racially/ethnic minoritised people with multiple long-term conditions: a systematic review and narrative synthesis
Source: BMC Public Health. 2026 Jun 19;26:2295. doi: 10.1186/s12889-026-28056-y (PMC13445657; doi:10.1186/s12889-026-28056-y)
Supplement: Supplementary file 1 [file 12889_2026_28056_MOESM1_ESM.pdf]

Pubmed search -20/02

Pubmed	Concept	Results
1	Healthcare	979,937
2	Interventions	3,437,096
3	Ethnicity	3,914,637
4	Mutiple Health Conditions	323,505
1+2+3+4		308
5	Inequalities	165,850
1+2+3+4+5		36

Concept A - Healthcare

“Health Personnel”[Mesh] OR “Healthcare provider”[Title/Abstract] OR “Health care provider”[Title/Abstract] OR “healthcare team”[Title/Abstract] OR “health care team”[Title/Abstract] OR “healthcare organisation”[Title/Abstract] OR “health care organisation”[Title/Abstract] OR “healthcare organization”[Title/Abstract] OR “health care organization”[Title/Abstract] OR “health personnel”[Title/Abstract] OR “health professional”[Title/Abstract] OR “healthcare staff”[Title/Abstract] OR “health care staff”[Title/Abstract] OR “health system”[Title/Abstract] OR “nurse”[Title/Abstract] OR “physician”[Title/Abstract] OR “doctor”[Title/Abstract] OR “family physician”[Title/Abstract] OR “allied health”[Title/Abstract]

Concept B – Intervention

“project*”[Title/Abstract] OR “intervention*”[Title/Abstract] OR “program*”[Title/Abstract] OR “programme*”[Title/Abstract] OR “case”[Title/Abstract] OR “case stud*”[Title/Abstract] OR “initiative*”[Title/Abstract] OR “audit”[Title/Abstract] OR “evaluation*”[Title/Abstract] OR “evaluative”[Title/Abstract] OR “research”[Title/Abstract] OR “comparison*”[Title/Abstract] OR “comparator*”[Title/Abstract] OR “study”[Title/Abstract] OR “Randomised controlled trial”[Title/Abstract] OR “Randomized controlled trial”[Title/Abstract] OR “Controlled clinical trial”[Title/Abstract] OR “Clinical trial”[Title/Abstract] OR “Placebo”[Title/Abstract] OR “randomly”[Title/Abstract] OR “trial”[Title/Abstract] OR “match”[Title/Abstract] NOT “animals”[Mesh]

Concept C – Ethnicity

"Ethnicity"[MeSH Terms] OR "Arabs"[MeSH Terms] OR "africa*"[Title/Abstract] OR "afro*"[Title/Abstract] OR "Asian"[Title/Abstract] OR "Asylum seeker"[Title/Abstract] OR "BME"[Title/Abstract] OR "BAME"[Title/Abstract] OR "bangladesh*"[Title/Abstract] OR "Black"[Title/Abstract] OR "Caribbean"[Title/Abstract] OR "China"[Title/Abstract] OR "Chinese"[Title/Abstract] OR "cultur*"[Title/Abstract] OR "divers*"[Title/Abstract] OR "Ethnic group"[Title/Abstract] OR "ethnic*"[Title/Abstract] OR "Gypsy"[Title/Abstract] OR "india*"[Title/Abstract] OR "Irish"[Title/Abstract] OR "Migrant"[Title/Abstract] OR "minorit*"[Title/Abstract] OR "Mixed"[Title/Abstract] OR "mixed ethnic*"[Title/Abstract] OR "multiple ethnic*"[Title/Abstract] OR "multirac*"[Title/Abstract] OR "multi rac*"[Title/Abstract] OR "Other White"[Title/Abstract] OR "pakistan*"[Title/Abstract] OR "Roma"[Title/Abstract] OR "White Other"[Title/Abstract] OR "Refugees"[MeSH Terms] OR "race"[Title/Abstract] OR "racial*"[Title/Abstract] OR "South Asian"[Title/Abstract] OR "White"[Title/Abstract]

Concept D – Inequalities

"Health Inequities"[MeSH Terms] OR "Health Status Disparities"[MeSH Terms] OR "Socioeconomic Disparities in Health"[MeSH Terms] OR "Health Equity"[MeSH Terms] OR "healthcare disparit*"[MeSH Terms] OR "inequalit*"[Title/Abstract] OR "disparit*"[Title/Abstract] OR "inequit*"

Concept E – Multiple health conditions

“Multiple Chronic Conditions”[Mesh] OR “Multimorbidity”[Mesh] OR “Comorbidity”[Mesh] OR “Comorbid*”[Title/Abstract] OR “Multipatholog*”[Title/Abstract] OR “multiple condition*”[Title/Abstract] OR “Multiple health conditions”[Title/Abstract] OR “Multiple health problems”[Title/Abstract] OR “Multiple medical conditions”[Title/Abstract] OR “Multiple medical problems”[Title/Abstract] OR “Pluripatholog*”[Title/Abstract] OR “Polymorbid*”[Title/Abstract] OR “multiple illness*”[Title/Abstract] OR “Multiple Chronic Health Conditions”[Title/Abstract] OR “Multiple Chronic Medical Conditions”[Title/Abstract] OR “multiple chronic illness*”[Title/Abstract]
