# Supplementary material for: Healthcare interventions to improve health outcomes for racially/ethnic minoritised people with multiple long-term conditions: a systematic review and narrative synthesis
Source: BMC Public Health. 2026 Jun 19;26:2295. doi: 10.1186/s12889-026-28056-y (PMC13445657; doi:10.1186/s12889-026-28056-y)
Supplement: Supplementary file 2 [file 12889_2026_28056_MOESM2_ESM.pdf]

**EPPI reviewer 6 RISK OF BIAS (COCHRANE)**<br>Flaws in the design, conduct, analysis, and reporting of randomised trials can cause the effect of an intervention to be underestimated or overestimated. The Cochrane Collaboration's tool for assessing risk of bias aims to make the process clearer and more accurate			
Domain	Subdomain	Description	Options
Selection bias	Random Sequence generation	Describe the method used to generate the allocation sequence in sufficient detail to allow an assessment of whether it should produce comparable groups	Low Risk<br>High Risk<br>Unclear
	Allocation Concealment	Describe the method used to conceal the allocation sequence in sufficient detail to determine whether intervention allocations could have been foreseen before or during enrolment	Low Risk<br>High Risk<br>Unclear
Performance bias	Blinding of participants and personnel	Describe all measures used, if any, to blind trial participants and researchers from knowledge of which intervention a participant received. Provide any information relating to whether the intended blinding was effective<br>*Assessments should be made for each main outcome or class of outcomes	Low Risk<br>High Risk<br>Unclear
Detection bias	Blinding of Outcome assessment*	Describe all measures used, if any, to blind outcome assessment from knowledge of which	Low Risk<br>High Risk<br>Unclear

		intervention a participant received. Provide any information relating to whether the intended blinding was effective *Assessments should be made for each main outcome or class of outcomes	
Attrition bias	Incomplete Outcome data	Describe the completeness of outcome data for each main outcome, including attrition and exclusions from the analysis. State whether attrition and exclusions were reported, the numbers in each intervention group (compared with total randomised participants), reasons for attrition or exclusions where reported, and any re-inclusions in analyses for the review *Assessments should be made for each main outcome or class of outcomes	Low Risk<br>High Risk<br>Unclear
Reporting Bias	Selective reporting	State how selective outcome reporting was examined and what was found	Low Risk<br>High Risk<br>Unclear
Other Bias	Anything Else ideally prespecified	State any important concerns about bias not covered in the other domains in the tool	Low Risk<br>High Risk<br>Unclear
