# Supplementary material for: Healthcare interventions to improve health outcomes for racially/ethnic minoritised people with multiple long-term conditions: a systematic review and narrative synthesis
Source: BMC Public Health. 2026 Jun 19;26:2295. doi: 10.1186/s12889-026-28056-y (PMC13445657; doi:10.1186/s12889-026-28056-y)
Supplement: Supplementary file 3 [file 12889_2026_28056_MOESM3_ESM.pdf]

Quality Assessment for Pre-Test/Post-Test Studies

	Fedder (2003)	Taufique (2023)	Zimbudzi (2023)	Darby (2024)
Study Quality Assessment Tools | NHLBI, NIH<br>**Pre-Test/Post Test study with no control group**	**Yes/No/Unclear/N/A**			
1. Was the study question or objective clearly stated?	Yes	Yes	Yes	Yes
2. Were eligibility/selection criteria for the study population prespecified and clearly described?	Yes	Yes	Yes	Yes
3. Were the participants in the study representative of those who would be eligible for the test/service/intervention in the general or clinical population of interest?	Unclear	Yes	Yes	Yes
4. Were all eligible participants that met the prespecified entry criteria enrolled?	Yes	Yes	Yes	Yes
5. Was the sample size sufficiently large to provide confidence in the findings?	Yes	No	Yes	No
6. Was the test/service/intervention clearly described and delivered consistently across the study population?	Yes	Yes	Yes	Yes
7. Were the outcome measures prespecified, clearly defined, valid, reliable, and assessed consistently across all study participants?	Yes	Yes	Yes	Yes
8. Were the people assessing the outcomes blinded to the participants' exposures/interventions?	No	Unclear	Unclear	Yes
9. Was the loss to follow-up after baseline 20% or less? Were those lost to follow-up accounted for in the analysis?	Unclear	Yes	Yes	Yes
10. Did the statistical methods examine changes in outcome measures from before to after the intervention? Were statistical tests done that provided p values for the pre-to-post changes?	Yes	Unclear	Yes	Yes
11. Were outcome measures of interest taken multiple times before the intervention and multiple times after the intervention (i.e., did they use an interrupted time-series design)?	No	Yes	Yes	No
12. If the intervention was conducted at a group level (e.g., a whole hospital, a community, etc.) did the statistical analysis take into account the use of	N/A	N/A	N/A	N/A

individual-level data to determine effects at the group level?				
**Quality Rating (Good, Fair, or Poor)**	Poor	Poor	Fair	Fair

Note: We did not specify a quality assessment tool to be used to assess pre-test/post-test studies in our protocol. We selected the NHBLI assessment tool for its clarity and ease of use.
