# Supplementary material for: Healthcare interventions to improve health outcomes for racially/ethnic minoritised people with multiple long-term conditions: a systematic review and narrative synthesis
Source: BMC Public Health. 2026 Jun 19;26:2295. doi: 10.1186/s12889-026-28056-y (PMC13445657; doi:10.1186/s12889-026-28056-y)

PRISMA Flow of Studies for searches conducted in August 2024 and 2025

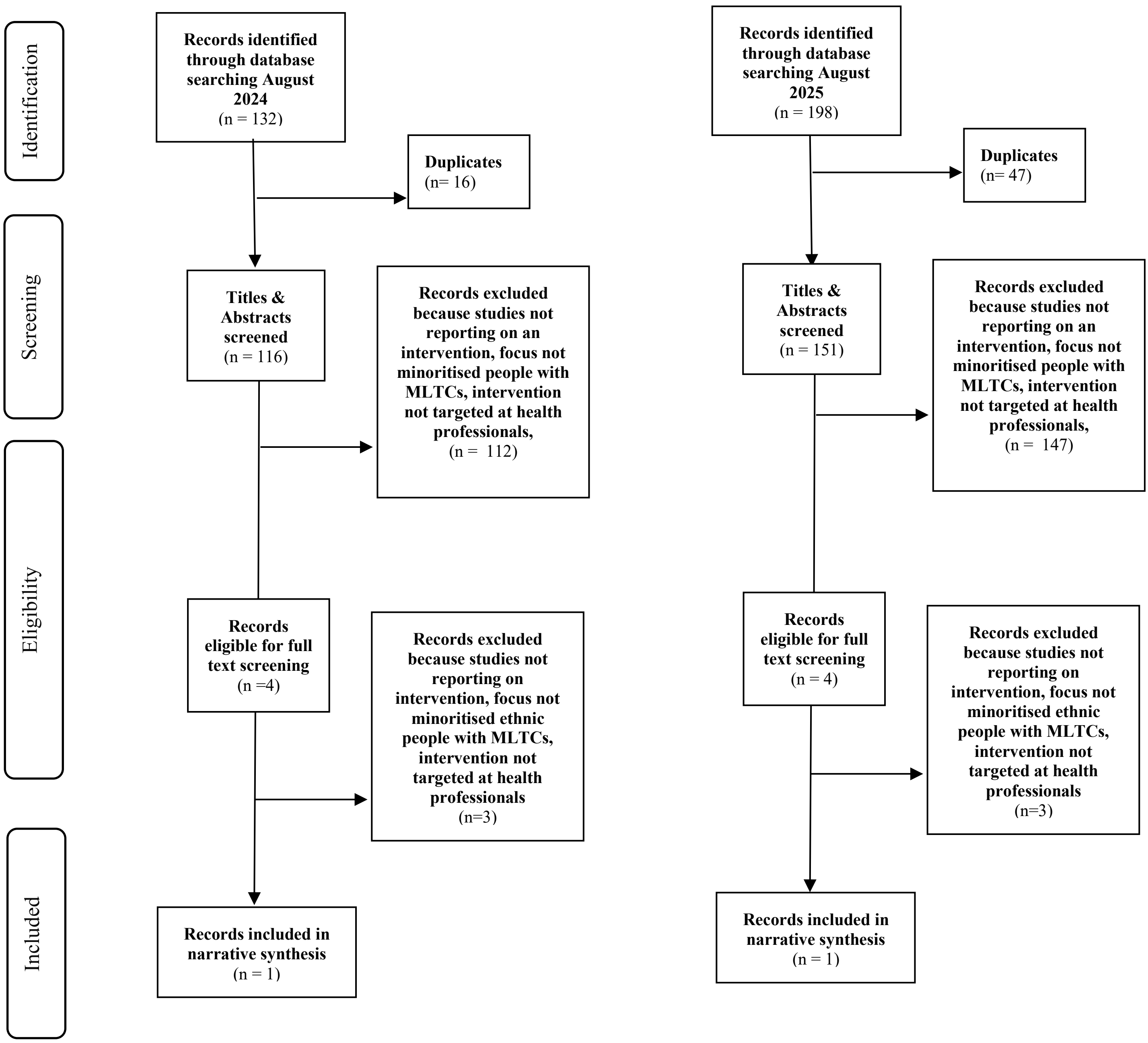

Supplement: Supplementary file 4 [file 12889_2026_28056_MOESM4_ESM.pdf]
